# Safety and pharmacokinetics of veliparib extended‐release in patients with advanced solid tumors: a phase I study

**DOI:** 10.1002/cam4.1488

**Published:** 2018-05-07

**Authors:** Theresa L. Werner, Jasgit Sachdev, Elizabeth M. Swisher, Martin Gutierrez, Muaiad Kittaneh, Mark N. Stein, Hao Xiong, Martin Dunbar, Danielle Sullivan, Philip Komarnitsky, Mark McKee, Antoinette R. Tan

**Affiliations:** ^1^ Huntsman Cancer Institute University of Utah Salt Lake City Utah; ^2^ HonorHealth Research Institute Scottsdale Arizona; ^3^ University of Washington/Seattle Cancer Care Alliance Seattle Washington; ^4^ John Theurer Cancer Center‐Hackensack University Medical Center Hackensack New Jersey; ^5^ Loyola University Chicago Maywood Illinois; ^6^ Rutgers Cancer Institute of New Jersey New Brunswick New Jersey; ^7^ AbbVie Inc. North Chicago Illinois

**Keywords:** *BRCA*, breast carcinoma, extended‐release formulation, ovarian carcinoma, PARP inhibitor, veliparib

## Abstract

The poly(ADP‐ribose) polymerase‐1/2 inhibitor veliparib is active against tumors deficient in homologous DNA damage repair. The pharmacokinetics and safety of veliparib extended‐release (ER) were evaluated in patients with advanced solid tumors. This phase I study assessed veliparib‐ER up to 800 mg once daily or 600 mg twice daily. Dose‐limiting toxicities (DLTs), recommended phase II dose (RP2D), and maximum tolerated dose (MTD) were assessed in cycle 1 and safety/tolerability during continuous administration (28‐day cycles). Seventy‐one patients (*n* = 53 ovarian, *n* = 17 breast, *n* = 1 prostate carcinoma) received veliparib; 50 had deleterious breast cancer susceptibility (*BRCA)* gene mutations. Single‐dose veliparib‐ER 200 mg (fasting) led to 58% lower peak concentration and similar area under the concentration‐time curve compared with veliparib immediate‐release (IR). Three patients experienced DLTs (grade 2: asthenia; grade 3: nausea/vomiting, seizure). RP2D and MTD for veliparib‐ER were 400 mg BID. The most frequent adverse events (AEs) were nausea (78.9%) and vomiting (50.7%). The most common grade 3/4 treatment‐related AEs were as follows: thrombocytopenia (7.0%), nausea, and anemia (4.2% each). Overall, 12 (27.3%) patients with ovarian and 10 (62.5%) patients with breast carcinoma had a partial response. Veliparib‐ER, versus veliparib‐IR, exhibited an improved pharmacokinetic profile and was well tolerated in patients with ovarian and *BRCA*‐mutated breast cancers.

## Introduction

Poly(ADP‐ribose) polymerase (PARP)‐1 and PARP‐2 are nuclear enzymes found in eukaryotes and facilitate DNA damage repair, thus contributing to the maintenance of genomic stability in normal cells 1, 2. In cancer cells, elevated levels of PARP have been linked to drug resistance and increased ability to withstand genotoxic stress 3. The dependency of neoplastic cells on PARP for DNA repair can be exploited therapeutically, as effective inhibition of PARP leads to the accumulation of single‐strand DNA breaks and ultimately results in double‐strand breaks that require repair by homologous recombination 2. Deficiencies in the capacity of homologous recombination to correct double‐strand breaks in many cancer types provide the rationale for the development of novel PARP inhibitors able to exploit this vulnerability.

Veliparib (ABT‐888) is a potent, orally bioavailable PARP‐1/2 inhibitor that has demonstrated clinical activity as a single agent in breast and ovarian carcinomas deficient in homologous recombination due to deleterious germline mutations of the breast cancer susceptibility (*BRCA*) genes 4, 5. In a phase I study, the recommended phase II dose (RP2D) of single‐agent veliparib was established at 400 mg twice‐daily (BID), with nausea and vomiting emerging as the predominant toxicity leading to dose delays and reductions 5. The pharmacokinetic (PK) profile of veliparib is characterized by high oral bioavailability, rapid absorption, and predominant elimination through urinary excretion of the unchanged drug 6. The administration of the immediate‐release (IR) formulation of veliparib (veliparib‐IR) in a BID schedule resulted in a peak‐to‐trough concentration ratio of approximately four.

We hypothesized that an extended‐release (ER) formulation of veliparib has the potential to lower the peak‐to‐trough ratio while maintaining exposure and improving tolerability. Thus, three ER formulations of veliparib (veliparib‐ER) were developed to investigate the potential for improved tolerability. The primary objective of this phase I study was to evaluate the oral bioavailability of three different veliparib‐ER formulations, to compare the findings with the current veliparib‐IR formulation, to assess the potential impact of food on the oral bioavailability of these formulations, and to establish the maximum tolerated dose (MTD), RP2D, and dosing schedule for the selected ER formulation. Secondary objectives were to evaluate the safety and tolerability, and exploratory efficacy of the veliparib‐ER formulations.

## Materials and Methods

### Study design

**Figure 1:**
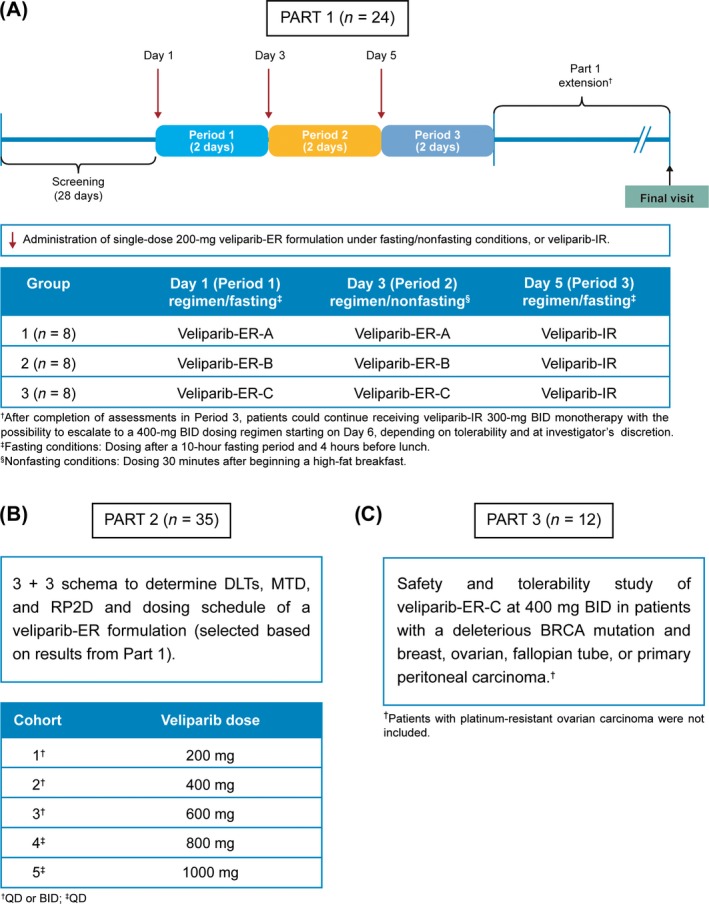
Study design for (A) Part 1, (B) Part 2, and (C) Part 3 of the study. BID, twice daily; *BRCA*, breast cancer susceptibility gene 1 or 2 (*BRCA 1* or *BRCA 2*); DLT, dose‐limiting toxicity; ER, extended‐release; ER‐A, ‐B, ‐C, one 200‐mg extended‐release tablet, formulation A, B, or C, respectively; IR, two 100‐mg immediate‐release capsules; MTD, maximum tolerated dose; QD, once daily; RP2D, recommended phase II dose.

This phase I multicenter study (NCT01853306) was initiated in 2013 and conducted in three parts. Part 1 was an open‐label, parallel‐group, three‐period, 6‐day crossover study to evaluate food effect and the relative bioavailability of three different veliparib‐ER formulations, using the current veliparib‐IR formulation as a reference. Initially, all patients enrolled in Part 1 of the study underwent a 28‐day screening period. After screening was finalized, patients were randomly assigned to one of the three treatment groups: Patients received a single dose consisting of one veliparib‐ER 200‐mg tablet, in formulations A (group 1), B (group 2), or C (group 3) (Fig. 1A). Each group of patients received only one of the three veliparib‐ER formulations. Patients from each group received one tablet of the predefined veliparib‐ER formulation (A, B, or C), administered under the fasting state on day 1 and, after a 48‐hour washout interval, each group of patients received the same veliparib‐ER formulation under the fed state on day 3. After another 48‐hour washout interval, all patients (groups 1, 2, and 3) received a single dose of veliparib consisting of two 100‐mg veliparib‐IR capsules, under fasting conditions on day 5 (Fig. 1A). After completion of assessments in Period 3, patients could continue to receive veliparib‐IR 300‐mg BID monotherapy with the possibility of escalating to a 400‐mg BID dosing regimen (Part 1 extension) starting on day 6. Escalation was dependent on tolerability and the investigator's discretion.

Part 2 was a dose‐escalation study conducted according to a 3 + 3 design to determine dose‐limiting toxicities (DLTs), the MTD, and the RP2D and dosing schedule of a veliparib‐ER formulation selected on the basis of the results from Part 1 (Fig. 1B), with veliparib continuously administered in 28‐day cycles. DLTs were determined during the first cycle. Hematologic and nonhematologic toxicities were considered as DLTs if deemed related to veliparib. Hematologic DLTs were as follows: grade 4 absolute neutrophil count (ANC) for >7 days (ANC <0.5 × 10^9^/L); grade 4 thrombocytopenia (platelets <25.0 × 10^9^/L); grade ≥3 ANC (ANC <1.0 × 10^9^/L) with fever defined as body temperature ≥38.5°C; and grade ≥3 decreased hemoglobin (<80 g/L). Nonhematologic DLTs were as follows: any grade >3 toxicity characterized by a ≥2‐grade increase from baseline possibly related to veliparib, excluding suboptimal treatment for nausea and vomiting; confirmed seizures of any grade; and grade 2 toxicity characterized by a ≥2‐grade increase from baseline and requiring dose adjustment or delay of >1 week. The MTD was defined as the highest veliparib dose level and schedule (once daily [QD] or BID) at which zero of three or one of six patients experienced a DLT. After completion of DLT assessments, patients could continue to receive veliparib‐ER at the same dose level and schedule tolerated, or switch to veliparib‐IR 300‐mg BID monotherapy with the possibility of escalating to 400 mg BID at the investigator's discretion.

Part 3 was a safety and tolerability study of the veliparib‐ER formulation administered continuously in 28‐day cycles in patients with a deleterious germline *BRCA* mutation and breast, ovarian, fallopian tube, or primary peritoneal carcinoma treated at the RP2D and dosing schedule determined from Part 2 (Fig. 1C). Patients could continue receiving veliparib‐ER at the same dose level until radiologic or clinical disease progression.

The trial was registered with the ClinicalTrials.gov registry (NCT01853306) and was approved by appropriate independent ethics committees/institutional review boards prior to initiation. The study was performed in accordance with the 1964 Declaration of Helsinki and its later amendments, with written informed consent obtained from all patients before study enrollment.

### Patients

Patients aged ≥18 years with an Eastern Cooperative Oncology Group (ECOG) performance status ≤2 and adequate bone marrow, renal, and hepatic functions were eligible on the basis of the following confirmed diagnoses: Parts 1 and 2, histologically or cytologically confirmed metastatic or unresectable malignancy for which no curative or therapeutic alternative exists *and* with a deleterious germline *BRCA* mutation, *or* high‐grade serous ovarian, fallopian tube, or peritoneal carcinoma; Part 3, histologically or cytologically confirmed metastatic or unresectable breast, ovarian, fallopian tube, or peritoneal carcinoma for which no curative or therapeutic alternative exists *and* with a deleterious *BRCA* mutation, and three or fewer prior chemotherapies in the metastatic setting, and evaluable disease according to Response Evaluation Criteria In Solid Tumors (RECIST) version 1.1 or Gynecologic Cancer Intergroup cancer antigen 125 criteria. Patients who received previous treatment with another PARP inhibitor were eligible for enrollment. Patients who received any anticancer hormonal therapy within 1 week before the first administration of study drug were excluded, as were patients with uncontrolled and clinically significant medical conditions, or patients who received previous treatment with veliparib. In addition, patients in Part 1 who received medications that were strong cytochrome P450 (CYP) 3A, 1A1, 2D6, or 2C19 inducers or inhibitors within 3 days or five half‐lives (whichever was the shortest) before the first administration of study drug were excluded. In Part 3, patients with platinum‐resistant/refractory ovarian carcinoma, defined as <6 months’ progression‐free survival from the completion of platinum‐based chemotherapy, were excluded, along with any patient with a history of surgical procedures preventing adequate gastrointestinal motility, pH, or absorption.

### Assessments

Blood samples for PK analysis were collected at the following time points: Part 1: 0 h (predose), and 0.5, 1, 2, 4, 6, 8, 10, 12, 16, 24, 28, and 48 h postdose on day 1; at 0.5, 1, 2, 4, 6, 8, 10, 12, 24, 28, and 48 h postdose on day 3; and at 0.5, 1, 2, 4, 6, 8, 10, and 12 h postdose on day 5. Part 2: on any day between days 3–8 at 0 h (predose), and at 1, 2, 4, 6, 8, 10, and 24 h postdose for QD dosing; and at 0 h (predose) and 1, 2, 4, 6, 8, 10, 12, and 24 h postdose for BID dosing. Part 3: 0 h (predose) on day 15 of cycle 1; and on day 1 of cycles 2, 3, and 4 (BID dosing). Noncompartmental methods were used to determine values for veliparib PK parameters. Adverse event (AE) severity was assessed according to the National Cancer Institute Common Terminology Criteria for Adverse Events (NCI CTCAE) version 4.0. Tumor evaluations were performed at screening, every 8 weeks after the first administration of study drug, and at the final visit using radiographic measurements from computed tomography or magnetic resonance imaging. Objective tumor responses were evaluated using RECIST version 1.1 criteria. Patients who received one or more doses of veliparib were included in the PK and safety analyses, as well as for evaluation of time to disease progression (TTP). Patients with one or more measurable lesion at baseline were included in the analysis of objective responses.

## Results

### Patients

A total of 71 patients were enrolled and treated with veliparib. In Part 1, 24 patients were randomized, including eight patients per treatment group. In Part 2, 35 consecutive patients were assigned to the veliparib‐ER formulation C (veliparib‐ER‐C) dosing regimen as follows: 200 mg QD (*n* = 4); 200 mg BID (*n* = 4); 400 mg QD (*n* = 4); 400 mg BID (*n* = 9); 600 mg QD (*n* = 6); 600 mg BID (*n* = 5); and 800 mg QD (*n* = 3). In Part 3, 12 patients with *BRCA*‐mutated breast or ovarian carcinomas received veliparib‐ER 400 mg BID in 28‐day cycles of continuous dosing. At the time of analysis, 71 (100%) patients had discontinued due to the following reasons: disease progression (*n* = 54), AEs related to progression (*n* = 9), AEs not related to progression (*n* = 7), consent withdrawal (*n* = 3), study discontinuation (*n* = 2), loss to follow‐up (*n* = 1), or other (*n* = 5). Patient demographics and baseline characteristics are summarized in Table 1. Patients were predominantly female (95.8%), aged ≥40 years (94.4%), with ECOG performance status 0 (69.0%). All patients were pretreated, with a median of four previous regimens (range, 1–10). The numbers of patients with breast or ovarian carcinoma who had a *BRCA* mutation or were sensitive, refractory, or resistant to platinum therapy are detailed in Table S1. Patients with platinum‐resistant/refractory ovarian carcinoma were included in the study (in Parts 1 and 2 only). The primary malignancies in the majority of patients were ovarian (53 [74.6%]) and breast (17 [23.9%]). Overall, 58 of 71 (81.7%) patients received prior platinum‐based regimens. Of these, six (10.3%) had breast carcinoma, and 52 (89.7%) had ovarian carcinoma. Overall, 50 of 71 (70.4%) had a *BRCA* mutation, 11 (15.5%) had no *BRCA* mutation, and 10 (14.1%) had an unknown BRCA status. In the population of patients with measurable disease at baseline (*N* = 60), 48 of 60 (80.0%) patients received prior platinum‐based regimens. Of these, five (10.4%) had breast carcinoma, and 43 (89.6%) had ovarian carcinoma; 30 (62.5%) had a *BRCA* mutation, nine (18.8%) had no *BRCA* mutation, and nine (18.8%) had an unknown BRCA status (Table S2).

**Table 1:** Patient demographics and baseline characteristics

Demographics and patient characteristics	Total (*N* = 71)
Females, *n* (%)	68 (95.8)
Age, years, *n* (%)	
<40	4 (5.6)
40 to <60	34 (47.9)
≥60	33 (46.5)
Race, *n* (%)	
White	66 (93.0)
Black	3 (4.2)
Asian	2 (2.8)
Number of prior regimens, median (range)	4 (1–10)
ECOG performance status, *n* (%)	
0	49 (69.0)
1	19 (26.8)
2	3 (4.2)

Baseline cancer characteristics	Part 1 (*n* = 24)	Part 2 (*n* = 35)	Part 3 (*n* = 12)	Total (*N* = 71)
Primary cancer type, *n* (%)				
Ovarian^a^	16 (66.7)	29 (82.9)	8 (66.7)	53 (74.6)
Breast	7 (29.2)	6 (17.1)	4 (33.3)	17 (23.9)
Prostate	1 (4.2)	0	0	1 (1.4)
Germline *BRCA* status,^b^ *n* (%)				
Mutation	17 (70.8)	21 (60.0)	12 (100)	50 (70.4)
No mutation	4 (16.7)	7 (20.0)	0	11 (15.5)
Unknown	3 (12.5)	7 (20.0)	0	10 (14.1)

*BRCA*, breast cancer susceptibility gene 1 or 2 (*BRCA1* or *BRCA2*); ECOG, Eastern Cooperative Oncology Group.

a Ovarian carcinoma included fallopian cancer, fallopian tube cancer, and fallopian tube carcinoma.

b Deleterious mutation.

### Pharmacokinetics

**Figure 2:**
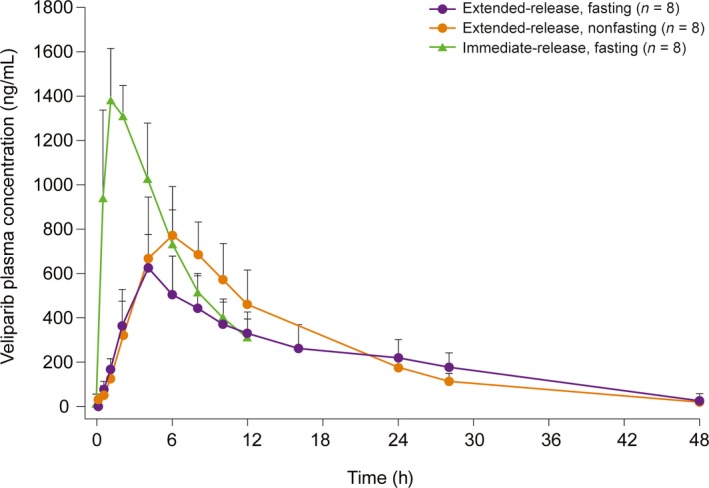
Veliparib concentration–time profiles after single‐dose administration of 200‐mg veliparib‐ER‐C or veliparib‐IR. Data are means + standard deviation. ER, extended‐release; IR, immediate‐release.

In Part 1, veliparib‐ER‐C showed the most preferred PK profile when compared with veliparib‐ER formulations A and B, on the basis of the observed maximum plasma concentration (*C*_max_), area under the concentration–time curve (AUC), and apparent half‐life values (data not shown). When compared with veliparib‐IR, administration of single‐dose veliparib‐ER‐C 200 mg under fasting conditions resulted in a 2.5‐h longer median time to *C*_max_ (*T*_max_), a 58% lower *C*_max_, and similar AUC from time 0 to infinity (AUC_∞_) (Fig. 2). Food had no significant effect on AUC_∞_ of veliparib‐ER‐C, but resulted in a moderate increase in *C*_max_ by 42%, and an increase in median *T*_max_ by 2.0 h compared with the fasting condition (Table 2). The relative bioavailability and 90% confidence intervals after single‐dose administration of 200‐mg veliparib‐ER‐C or veliparib‐IR are presented in Table S3. In Part 2, the administration of veliparib‐ER‐C QD and BID resulted in a median steady‐state peak‐to‐trough concentration ratio of 4.4 and 2.0, respectively, across dose levels. Compared with veliparib‐ER‐C 400 mg QD, the 200‐mg BID regimen maintained higher observed minimum plasma concentration (*C*_min_) and lower *C*_max_ at steady state (Table S4).

**Table 2:** Pharmacokinetic parameters after single‐dose administration of 200‐mg veliparib‐ER‐C or veliparib‐IR

Pharmacokinetic parameters	Veliparib‐ER‐C (fasting)	Veliparib‐ER‐C (nonfasting)	Veliparib‐IR (fasting)
*N*	8	8	8
*C*_max_, μg/mL^a^	0.615 (23)	0.876 (14)	1.460 (11)
*T*_max_, h^b^	4.0 (2.0–4.0)	6.0 (4.0–10.0)	1.5 (0.5–4.0)
*t*_1/2_, h^c^	8.2 ± 3.0	7.3 ± 1.7	4.9 ± 1.4
AUC_*t*_, μg h/mL^a^	10.1 (30)	12.4 (29)	8.96 (12)
AUC_∞_, μg h/mL^a^	11.1 (28)	12.5 (28)	11.4 (23)

AUC_∞_, area under the plasma concentration–time curve from time 0 to infinity; AUC_*t*_, area under the plasma concentration–time curve from time 0 to time of the last measurable concentration; *C*_max_, observed maximum plasma concentration; *T*_max_, time to *C*_max_; *t*_1/2_, terminal phase elimination half‐life; veliparib‐ER‐C, veliparib extended‐release formulation C; veliparib‐IR, veliparib immediate‐release formulation.

a Geometric mean (% coefficient of variation).

b Median (minimum–maximum).

c Harmonic mean ± pseudostandard deviation.

### MTD and RP2D determination

Three patients in Part 2 experienced DLTs. In the veliparib‐ER‐C 600‐mg BID dose cohort, one patient had grade 2 asthenia (intolerable), and one patient had grade 3 nausea and vomiting during cycle 1 of treatment. In the veliparib‐ER‐C 800‐mg QD dose cohort, one patient had grade 3 seizure. The MTD and RP2D were therefore defined as 400 mg BID for the veliparib‐ER‐C formulation.

### Safety

The median duration of exposure to veliparib was 119 days (range, 2–1211 days). In Parts 1, 2, and 3, the median duration of exposure to veliparib was 81.5 days (range, 9–1120), 145 days (range, 2–1211), and 196 days (range, 55–1158), respectively. Sixty‐seven of 71 (94.4%) and 70 of 71 (98.6%) patients experienced one or more treatment‐emergent AE (TEAE) during cycle 1 and all cycles, respectively. Grade 3 or 4 TEAEs were reported by 13 of 71 (18.3%) and 28 of 71 (39.4%) patients during cycle 1 and all cycles, respectively (Table 3). Serious adverse events (SAEs) leading to veliparib‐ER dose interruptions or reductions were experienced by six or fewer (8.5%) patients during cycle 1 and ≤14 (19.7%) patients during all cycles. SAEs leading to veliparib‐ER discontinuation occurred in four (5.6%) patients in cycle 1 and eight (11.3%) patients during all cycles (Table 3). TEAEs occurring in ≥20% of patients during all treatment cycles are summarized in Table 4. Overall, the most frequent TEAEs were nausea (78.9%), vomiting (50.7%), constipation (32.4%), fatigue (32.4%), and diarrhea (32.4%). The most common grade 3 or 4 treatment‐related AEs (overall [%]; Part 1 extension/Part 2/Part 3) were thrombocytopenia (7.0%; 2/3/0), nausea (4.2%; 0/2/1), and anemia (4.2%; 0/3/0).

**Table 3:** Summary of treatment‐emergent adverse events and serious adverse events

TEAE, *n* (%)	Cycle 1				All cycles				Part 1, PK BA portion (*n* = 24)
	Part 1, extension (*n* = 24)	Part 2 (*n* = 35)	Part 3 (*n* = 12)	All patients (*N* = 71)	Part 1, extension (*n* = 24)	Part 2 (*n* = 35)	Part 3 (*n* = 12)	All patients (*N* = 71)	
Any AE	23 (95.8)	34 (97.1)	10 (83.3)	67 (94.4)	23 (95.8)	35 (100)	12 (100)	70 (98.6)	17 (70.8)
At least possibly related to study drug	14 (58.3)	29 (82.9)	7 (58.3)	50 (70.4)	17 (70.8)	33 (94.3)	10 (83.3)	60 (84.5)	7 (29.2)
NCI CTCAE grade 3 or 4	4 (16.7)	8 (22.9)	1 (8.3)	13 (18.3)	7 (29.2)	17 (48.6)	4 (33.3)	28 (39.4)	2 (8.3)
Any SAE	4 (16.7)	5 (14.3)	1 (8.3)	10 (14.1)	5 (20.8)	11 (31.4)	4 (33.3)	20 (28.2)	2 (8.3)
SAE leading to discontinuation	3 (12.5)	1 (2.9)	0	4 (5.6)	3 (12.5)	4 (11.4)	1 (8.3)	8 (11.3)	0
SAE leading to dose interruption	2 (8.3)	3 (8.6)	1 (8.3)	6 (8.5)	4 (16.7)	6 (17.1)	4 (33.3)	14 (19.7)	1 (4.2)
SAE leading to dose reduction	0	1 (2.9)	0	1 (1.4)	0	1 (2.9)	0	1 (1.4)	0

AE, adverse event; BA, bioavailability; NCI CTCAE, National Cancer Institute Common Terminology Criteria for Adverse Events; PK, pharmacokinetic; SAE, serious adverse event; TEAE, treatment‐emergent adverse event.

**Table 4:** Treatment‐emergent adverse events reported in ≥20% of patients

TEAE, *n* (%)	Part 1, PK phase (*n* = 24)	All cycles			Total (*N* = 71)
		Part 1, extension (*n* = 24)	Part 2 (*n* = 35)	Part 3 (*n* = 12)	
Nausea	4 (16.7)	12 (50.0)	31 (88.6)	9 (75.0)	56 (78.9)
Grade 1	4 (16.7)	11 (45.8)	24 (68.6)	6 (50.0)	45 (63.4)
Grade 2	0	3 (12.5)	5 (14.3)	2 (16.7)	10 (14.1)
Grade 3	0	0	2 (5.7)	1 (8.3)	3 (4.2)
Vomiting	1 (4.2)	11 (45.8)	17 (48.6)	7 (58.3)	36 (50.7)
Grade 1	1 (4.2)	11 (45.8)	13 (37.1)	4 (33.3)	29 (40.8)
Grade 2	0	0	3 (8.6)	2 (16.7)	5 (7.0)
Grade 3	0	0	1 (2.9)	1 (8.3)	2 (2.8)
Constipation	0	5 (20.8)	14 (40.0)	4 (33.3)	23 (32.4)
Fatigue	0	7 (29.2)	14 (40.0)	2 (16.7)	23 (32.4)
Diarrhea	2 (8.3)	5 (20.8)	10 (28.6)	6 (50.0)	23 (32.4)
Abdominal pain	3 (12.5)	3 (12.5)	7 (20.0)	4 (33.3)	17 (23.9)
Urinary tract infection	0	7 (29.2)	5 (14.3)	3 (25.0)	15 (21.1)
Decreased appetite	1 (4.2)	1 (4.2)	11 (31.4)	2 (16.7)	15 (21.1)
Anemia	1 (4.2)	3 (12.5)	10 (28.6)	2 (16.7)	16 (2.51)

PK, pharmacokinetic; TEAE, treatment‐emergent adverse event.

### Efficacy

The exploratory efficacy analysis of objective response rate (ORR) included 60 patients who received one or more dose of veliparib with one or more measurable lesion at baseline. The number of patients with measurable disease at baseline who received prior platinum‐based regimens is detailed per cancer type and BRCA status in Table S2. For TTP evaluation, all 71 patients who received one or more veliparib dose were included. The number of patients who received prior platinum‐based regimens is detailed for each study part and overall in Table S5. Among 44 patients with ovarian carcinoma who were evaluable for response, 12 (27.3%) had a partial response, including five (11.4%) confirmed partial responses (of which three were recorded in patients with a deleterious *BRCA* mutation) (Table 5). Among 16 evaluable patients with breast carcinoma (all *BRCA* mutation carriers), 10 (62.5%) had a partial response, including four (25.0%) confirmed partial responses (Table 5). Thus, an overall (confirmed and unconfirmed) ORR of 36.7% and a confirmed overall ORR of 15% were observed among the 60 evaluable patients. Among 42 evaluable breast and ovarian carcinoma patients with deleterious *BRCA* mutations, 17 (40.5%) had a partial response, including seven (16.7%) confirmed partial responses (three of 26 [11.5%] patients with ovarian carcinoma and four of 16 [25.0%] patients with breast carcinoma). Four of nine (44.4%) patients with unknown *BRCA* status and measurable disease at baseline had a partial response (two confirmed, one of whom had a BRCA‐associated RING domain protein 1 mutation; two unconfirmed), all of whom had ovarian carcinoma. No confirmed and one unconfirmed objective responses were observed in ovarian carcinoma patients with no *BRCA* mutation.

**Table 5:** Summary of confirmed tumor response, by ovarian or breast carcinoma type and overall^a^

	Ovarian (*n* = 44)^b^	Breast (*n* = 16)	Overall (*N* = 60)
ORR (CR + PR), *n*/*N* (%) (95% CI)	5 (11.4)—	4 (25.0)—	9/60 (15.0) (7.1–26.6)
CR, *n* (%)	0	0	0
PR, *n* (%)	5 (11.4)	4 (25.0)	9 (15.0)
**Deleterious** ***BRCA*** **mutation**			
	**Ovarian (** *n* ** = 26)**	**Breast (** *n* ** = 16)**	**Overall (** *N* ** = 42)**
ORR (CR + PR), *n*/*N* (%)	3 (11.5)	4 (25.0)	7/42 (16.7)
(95% CI)	—	—	(7.0–31.4)
CR, *n* (%)	0	0	0
PR, *n* (%)	3 (11.5)	4 (25.0)	7 (16.7)

*BRCA*, breast cancer susceptibility gene 1 or 2 (*BRCA1* or *BRCA2*); CR, complete response; ORR, objective response rate; PR, partial response.

a Only patients with one or more measurable lesion at baseline were included in the analysis.

b Ovarian carcinoma included fallopian cancer, fallopian tube cancer, and fallopian tube carcinoma.

For the overall population (*N* = 71), the median TTP was 149 days (95% CI, 64–186 days). TTP for patients with breast and ovarian carcinomas is shown in Fig. S1. The overall 6‐month TTP rate (i.e., had not progressed at 6 months) was 38.1% (95% CI, 26.0–50.1). For patients with ovarian (*n* = 53) and breast (*n* = 17) carcinomas, the overall 6‐month TTP rates were 31.8% (95% CI, 18.9–45.5) and 53.5% (95% CI, 25.9–74.8), respectively. Among patients with deleterious *BRCA* mutations, the overall 6‐month TTP rate was 43.0% (95% CI, 28.1–57.0), with 35.7% (95% CI, 18.9–53.0) of patients with ovarian carcinoma and 53.5% (95% CI, 25.9–74.8) of patients with breast carcinoma not having progression at 6 months. In comparison, the 6‐month TTP rates among patients without *BRCA* mutations and with unknown *BRCA* mutation status were 22.9% (95% CI, 3.5–52.2) and 27.8% (95% CI, 4.4–59.1), respectively.

## Discussion

PARP inhibitors are a promising therapeutic strategy in a range of cancer types 7, 8 and have demonstrated the highest activity in cancers of patients with deleterious *BRCA* mutations associated with homologous recombination pathway deficiency 4, 9, 10. Inhibition of PARP in cancers deficient in homologous recombination leads to the formation of double‐strand DNA breaks that cannot be accurately repaired, resulting in neoplastic cell death 11. In addition, the inhibition of PARP‐1 impairs DNA repair following radiation therapy or cytotoxic chemotherapy, while PARP inhibitors may also stabilize or “trap” PARP‐1 and PARP‐2 at sites of DNA damage to produce highly toxic complexes that lead to cell death 2. From a tolerability perspective, treatment with PARP inhibitors is commonly associated with dose interruptions, and a smaller proportion of patients require dose reduction and treatment discontinuation. The most common AEs leading to treatment discontinuation include nausea, vomiting, anemia, and thrombocytopenia 5, 6, 12, 13, 14, 15, 16.

In phase I and II clinical trials, veliparib administered as single‐agent therapy was well tolerated in patients with advanced solid tumors 4, 5. The most common all‐grade toxicities were nausea, vomiting, fatigue, and lymphopenia. Myelosuppression was modest, with only 2% of patients experiencing grade 3 or 4 neutropenia or thrombocytopenia. Similar to other PARP inhibitors, 78% of patients had dose modifications, predominantly for nausea and vomiting 4. Gastrointestinal toxicity primarily occurred in earlier treatment courses and was manageable through aggressive use of anti‐emetics, as well as through dose delays and reductions 2. Recently, preliminary results from two phase III clinical trials of veliparib combined with carboplatin and paclitaxel in patients with non‐small‐cell lung cancer (NCT02106546) or triple‐negative breast cancer (NCT02163694) indicated that the trials did not meet their primary endpoints of improvements in overall survival or in complete pathologic response following veliparib treatment 17. Nevertheless, as the AEs encountered in the phase I and II trials of veliparib are characteristic for other PARP inhibitors as well (e.g., olaparib, rucaparib) 13, 14, 15 and can be problematic for maintaining compliance with an orally administered dosing regimen, we sought to improve the tolerability profile of veliparib by adjusting the formulation to obtain an improved PK profile. Following screening of three potential ER formulations of veliparib, the veliparib‐ER‐C formulation was selected as the best candidate for further evaluation on the basis of its apparent half‐life. When compared with veliparib‐IR, the veliparib‐ER‐C formulation yielded a markedly lower *C*_max_ and prolonged *T*_max_. Food had no significant effect on total systemic exposure to veliparib‐ER‐C, but led to a moderate increase in both *C*_max_ and *T*_max_ compared with the fasted state. BID dosing of veliparib‐ER‐C yielded a steady‐state peak‐to‐trough concentration ratio superior to the BID dosing of veliparib‐IR, while QD dosing of veliparib‐ER‐C exhibited a ratio compared with the BID dosing of veliparib‐IR.

We next sought to establish the tolerability profile of the novel veliparib‐ER‐C formulation and to compare this with the conventional veliparib‐IR formulation. Veliparib‐ER‐C was shown to be well tolerated in patients with advanced solid tumors, with a RP2D of 400 mg BID, identical to that of veliparib‐IR in an earlier study 5. No clear differences in tolerability were observed for veliparib‐ER‐C compared with historical data from studies of veliparib‐IR 4, 5. Gastrointestinal toxicities in particular remained prominent, with nausea and vomiting of low grade occurring in approximately 80% and 50% of patients, respectively. This might indicate that the use of an ER formulation of veliparib may not provide significant tolerability benefits compared with the standard IR formulation.

Combined data from patients treated with veliparib‐ER‐C and veliparib‐IR formulations were evaluated for efficacy. In patients with breast or ovarian carcinomas with or without deleterious *BRCA* mutations, veliparib showed antitumor activity in terms of TTP. Antitumor activity of veliparib was also observed in patients with deleterious *BRCA* mutations in terms of objective response. Although data are preliminary due to the small sample size, the absence of a control group, and no formal hypothesis testing, patients with deleterious *BRCA* mutations appeared to have longer TTP compared with patients without a known BRCA mutation. These data are consistent with the known activity of PARP inhibitors in patients with deleterious *BRCA* mutations 4, 18, 19. Furthermore, as this was a phase I study, there were no limits on the prior number of therapies patients had received. In this setting, the relatively low response rate is explicable.

In conclusion, veliparib‐ER‐C showed reduced *C*_max_ versus the IR formulation. Veliparib‐ER‐C did not appear to show significant differences in tolerability and safety compared with veliparib‐IR; however, the study design complicates such comparisons. The improved PK profile of the veliparib‐ER‐C formulation warrants further investigation of QD dosing of veliparib‐ER‐C as an alternative to the BID dosing of veliparib‐IR.

## Conflict of Interest

T.L. Werner obtained research funding from AbbVie, Mirati Therapeutics, Novartis, Roche/Genentech, and Tesaro. J. Sachdev obtained research funding from Celgene, Pfizer and research support from Genentech. Advisory board: Celgene. Travel investigator meeting: Celgene. M. Gutierrez performed consulting or advisory role for Bayer and Lilly; speakers’ bureau for Bristol‐Myers Squibb and Merck Sharp & Dohme; stock and other ownership interests from COTA; research funding from AbbVie, Acceleron Pharma, Bristol‐Myers Squibb, Daiichi Sankyo, EMD Serono, Esanex, Gilead Sciences, Incyte, Karyopharm Therapeutics, Lilly, MedImmune, Novartis, Rexahn Pharmaceuticals, and TG Therapeutics. E. Swisher and M. Kittaneh declared no potential conflict of interests. M. Stein obtained research funding from AbbVie, Astellas Pharma, Bavarian Nordic, Janssen Oncology, Medivation/Astellas, Merck Sharp & Dohme, and Oncoceutics. H. Xiong, M. Dunbar, D. Sullivan, P. Komarnitsky, and M. McKee are employees and stakeholders of AbbVie Inc. A.R. Tan obtained research funding from AbbVie, Genentech, MedImmune, Merck Sharp & Dohme, and Pfizer. Travel to investigator meeting: Caris Life Sciences.

## Supporting information


**Table S1.** Number and percentage of patients per cancer type and overall, detailed per *BRCA* mutation status, number of prior platinum‐based regimens, and platinum sensitivity.**Table S2.** Numbers and percentages of patients with measurable disease who received prior platinum‐based regimens, per cancer type and *BRCA* mutation status, in each study part and overall.**Table S3.** Relative bioavailability and 90% confidence intervals after single‐dose administration of 200‐mg veliparib‐ER‐C or veliparib‐IR*.**Table S4.** Geometric mean (% CV) multiple‐dose pharmacokinetic parameters of veliparib‐ER‐C in Part 2 of the study.**Table S5.** Numbers and percentages of patients who received prior platinum‐based regimens, per study part and in the overall population.**Figure S1.** Time to disease progression for breast and ovarian carcinoma patients.
