# Supplementary material for: Safety and pharmacokinetics of veliparib extended‐release in patients with advanced solid tumors: a phase I study
Source: Cancer Med. 2018 May 7;7(6):2360–9. doi: 10.1002/cam4.1488 (PMC6010916; doi:10.1002/cam4.1488)

**Supporting information**

| **Supporting information table 1: Number and percentage of patients per cancer type and overall, detailed per *BRCA* mutation status, number of prior platinum-based regimens, and platinum sensitivity** | | | | |
| --- | --- | --- | --- | --- |
|  | **Breast**  **(n=17)** | **Ovarian**  **(n=53)** | **Prostate**  **(n=1)** | **Total**  **(N=71)** |
| **Germline BRCA status, n (%)**  Mutation  No mutation  Unknown | 17 (100.0)  0  0 | 32 (60.4)  11 (20.7)  10 (18.9) | 1 (100.0)  0  0 | 50 (70.4)  11 (15.5)  10 (14.1) |
| **Prior platinum-based regimens, n (%)**  0  1  2  3  4  ≥5 | 11 (64.7)  3 (17.6)  3 (17.6)  0  0  0 | 1 (1.9)  7 (13.2)  24 (45.3)  13 (24.5)  5 (9.4)  3 (5.7) | 1 (100.0)  0  0  0  0  0 | 13 (18.3)  10 (14.1)  27 (38.0)  13 (18.3)  5 (7.1)  3 (4.2) |
| **Platinum sensitivity, n (%)**  Sensitive  Refractory  Resistant  Unknown  NE | NE  NE  NE  NE  17 (100.0) | 18 (34.0)  14 (26.4)  18 (34.0)  3 (5.7)  0 | NE  NE  NE  NE  1 (100.0) | 18 (25.4)  14 (19.7)  18 (25.4)  3 (4.2)  18 (25.4) |
| Abbreviations: *BRCA*=breast cancer susceptibility gene 1 or 2 (*BRCA1* or *BRCA2*); NE=not evaluated. | | | | |

| **Supporting information table 2: Numbers and percentages of patients with measurable disease who received prior platinum-based regimens, per cancer type and *BRCA* mutation status, in each study part and overall** | | | | |
| --- | --- | --- | --- | --- |
|  | **Part 1**  **(n=20)** | **Part 2**  **(n=30)** | **Part 3**  **(n=10)** | **Total**  **(N=60)** |
| **Regimens, n (%)**  1  2  3  4  ≥5  NR | 3 (15.0)  6 (30.0)  5 (25.0)  0 (0.0)  2 (10.0)  4 (20.0) | 5 (16.7)  14 (46.7)  4 (13.3)  3 (10.0)  0 (0.0)  4 (13.3) | 1 (10.0)  3 (30.0)  2 (20.0)  0 (0.0)  0 (0.0)  4 (40.0) | 9 (15.0)  23 (38.3)  11 (18.3)  3 (5.0)  2 (3.3)  12 (20.0) |
| **Tumour type, n (%)**  **Breast**  1  2  3  4  ≥5  NR  **Ovarian**  1  2  3  4  ≥5  NR | 2 (10.0)  1 (5.0)  0 (0.0)  0 (0.0)  0 (0.0)  17 (85.0)  1 (5.0)  5 (25.0)  5 (25.0)  0 (0.0)  2 (10.0)  7 (35.0) | 1 (3.3)  1 (3.3)  0 (0.0)  0 (0.0)  0 (0.0)  28 (93.3)  4 (13.3)  13 (43.3)  4 (13.3)  3 (10.0)  0 (0.0)  6 (20.0) | 0 (0.0)  0 (0.0)  0 (0.0)  0 (0.0)  0 (0.0)  10 (100.0)  1 (10.0)  3 (30.0)  2 (20.0)  0 (0.0)  0 (0.0)  4 (40.0) | 3 (5.0)  2 (3.3)  0 (0.0)  0 (0.0)  0 (0.0)  55 (91.7)  6 (10.0)  21 (35.0)  11 (18.3)  3 (5.0)  2 (3.3)  17 (28.3) |
| **BRCA status**  **Mutation**  1  2  3  4  ≥5  NR  **No mutation**  1  2  3  4  ≥5  NR  **Unknown**  1  2  3  4  ≥5  NR | 2 (10.0)  5 (25.0)  2 (10.0)  0 (0.0)  1 (5.0)  10 (50.0)  1 (5.0)  0 (0.0)  2 (10.0)  0 (0.0)  0 (0.0)  17 (85.0)  0 (0.0)  1 (5.0)  1 (5.0)  0 (0.0)  1 (5.0)  17 (85.0) | 4 (13.3)  6 (20.0)  2 (6.7)  2 (6.7)  0 (0.0)  16 (53.3)  1 (3.3)  4 (13.3)  1 (3.3)  0 (0.0)  0 (0.0)  24 (80.0)  0 (0.0)  4 (13.3)  1 (3.33)  1 (3.33)  0 (0.0)  24 (80.0) | 1 (10.0)  3 (30.0)  2 (20.0)  0 (0.0)  0 (0.0)  4 (40.0)  0 (0.0)  0 (0.0)  0 (0.0)  0 (0.0)  0 (0.0)  10 (100.0)  0 (0.0)  0 (0.0)  0 (0.0)  0 (0.0)  0 (0.0)  10 (100.0) | 7 (11.7)  14 (23.3)  6 (10.0)  2 (3.33)  1 (1.7)  30 (50.0)  2 (3.3)  4 (6.7)  3 (5.0)  0 (0.0)  0 (0.0)  51 (85.0)  0 (0.0)  5 (8.3)  2 (3.3)  1 (1.7)  1 (1.7)  51 (85.0) |
| Abbreviations: *BRCA*=breast cancer susceptibility gene 1 or 2 (*BRCA1* or *BRCA2*); NR=none reported. | | | | |

| **Supporting information table 3: Relative bioavailability and 90% confidence intervals after single-dose administration of 200-mg veliparib-ER-C or veliparib-IR*** | | | | | |
| --- | --- | --- | --- | --- | --- |
| **Regimens**  **Test vs. reference** | **Pharmacokinetic parameters (units)** | **Central value^a^** | | **Relative bioavailability** | |
|  |  | **Test** | **Reference** | **Point**  **estimate^b^** | **90% Confidence interval^c^** |
| Veliparib-ER-C (fasting) vs. veliparib-IR  (fasting) | C_max_ (ng/mL) | 615 | 1460 | 0.420 | 0.372–0.475 |
|  | AUC_∞_ (ng•h/mL) | 11100 | 11400 | 0.967 | 0.875–1.068 |
| Veliparib-ER-C  (fed) vs. veliparib-ER-C (fasting) | C_max_ (ng/mL) | 876 | 615 | 1.424 | 1.261–1.609 |
|  | AUC_t_ (ng•h/mL) | 12400 | 10100 | 1.221 | 1.051–1.420 |
|  | AUC_∞_ (ng•h/mL) | 12500 | 11100 | 1.133 | 1.025–1.252 |
| Abbreviations: AUC_∞_=area under the concentration-time curve from time 0 to infinity; AUC_t_=area under the concentration-time curve from time 0 to time point t; C_max_=observed maximum plasma concentration; ER-C=extended release formulation C; IR=immediate release formulation; T_max_=maximum time to C_max_.  *Veliparib-ER-C: one 200-mg tablet; veliparib-IR: two 100-mg capsules.  ^a^Antilogarithm of the least square means for logarithms.  ^b^Antilogarithm of the difference (test minus reference) of the least square means for logarithms.  ^c^Antilogarithm of the endpoints of confidence intervals for the difference of logarithms means. | | | | | |

| **Supporting information table 4: Geometric mean (% CV) multiple-dose pharmacokinetic parameters of veliparib-ER-C in Part 2 of the study** | | | | | | | |
| --- | --- | --- | --- | --- | --- | --- | --- |
| **Pharmacokinetic parameters (units)** | **Veliparib administered QD** | | | | | | |
|  | **200 mg** | **400 mg** | | **600 mg** | | | **800 mg** |
| N | 4 | 4 | | 6 | | | 1 |
| C_max_ (μg/mL) | 0.831 (6) | 1.75 (27) | | 3.13 (37) | | | 4.84 |
| T_max_^†^ (h) | 4.0 (2.0–80) | 5.0 (4.0–8.0) | | 5.0 (1.3–8.0) | | | 6.0 |
| AUC_0-24_ (μg•h/mL) | 11.5 (2) | 24.6 (39) | | 51.7 (29) | | | 64.3 |
| C_min_ (μg/mL) | 0.179 (50) | 0.236 (101) | | 0.865 (51) | | | 0.724 |
| **Pharmacokinetic parameters (units)** | **Veliparib administered BID** | | | | |  | |
|  | **200 mg** | | **400 mg** | | **600 mg** |  | |
| N | 5 | | 8 | | 3 |  | |
| C_max_ (μg/mL) | 1.65 (75) | | 3.18 (33) | | 5.27 (25) |  | |
| T_max_^†^ (h) | 4.0 (0–6.0) | | 4.0 (2.0–6.0) | | 4.0 (2.0–4.0) |  | |
| AUC_0-12_ (μg•h/mL) | 14.8 (86) | | 28.7 (36) | | 51.6 (20) |  | |
| C_min_ (μg/mL) | 0.743 (103) | | 1.54 (40) | | 3.06 (14) |  | |
| Abbreviations: AUC_0-12_=area under the concentration-time curve from time 0 to 12 hours; AUC_0-24_=area under the concentration-time curve from time 0 to 24 hours; BID=twice-daily; C_max_=observed maximum plasma concentration; C_min_=observed minimum plasma concentration; CV=coefficient of variation; ER-C=extended release formulation C; T_max_=maximum time to C_max_; QD=once-daily.  Note: Pharmacokinetic samples were collected post-dose on a day between days 3–8 of cycle 1.  ^†^Median (minimum – maximum). | | | | | | | |

| **Supporting information table 5: Numbers and percentages of patients who received prior platinum-based regimens, per study part and in the overall population** | | | | |
| --- | --- | --- | --- | --- |
|  | **Part 1**  **(n=24)** | **Part 2**  **(n=35)** | **Part 3**  **(n=12)** | **Overall**  **(N=71)** |
| **Prior platinum therapy, n (%)**  Yes  No | 19 (79.2)  5 (20.8) | 31 (88.6)  4 (11.4) | 8 (66.7)  4 (33.3) | 58 (81.7)  13 (18.3) |

**Titles and legends to figures**

**Supporting information figure 1.** Time to disease progression for breast and ovarian carcinoma patients.

All patients dosed with veliparib were included. Group A: Breast – deleterious *BRCA*; Group B: Ovarian – deleterious *BRCA*; Group C: Ovarian – no deleterious *BRCA*; Group D – Ovarian – unknown *BRCA*. *BRCA*=breast cancer susceptibility gene 1 or 2 (*BRCA1* or *BRCA2*).


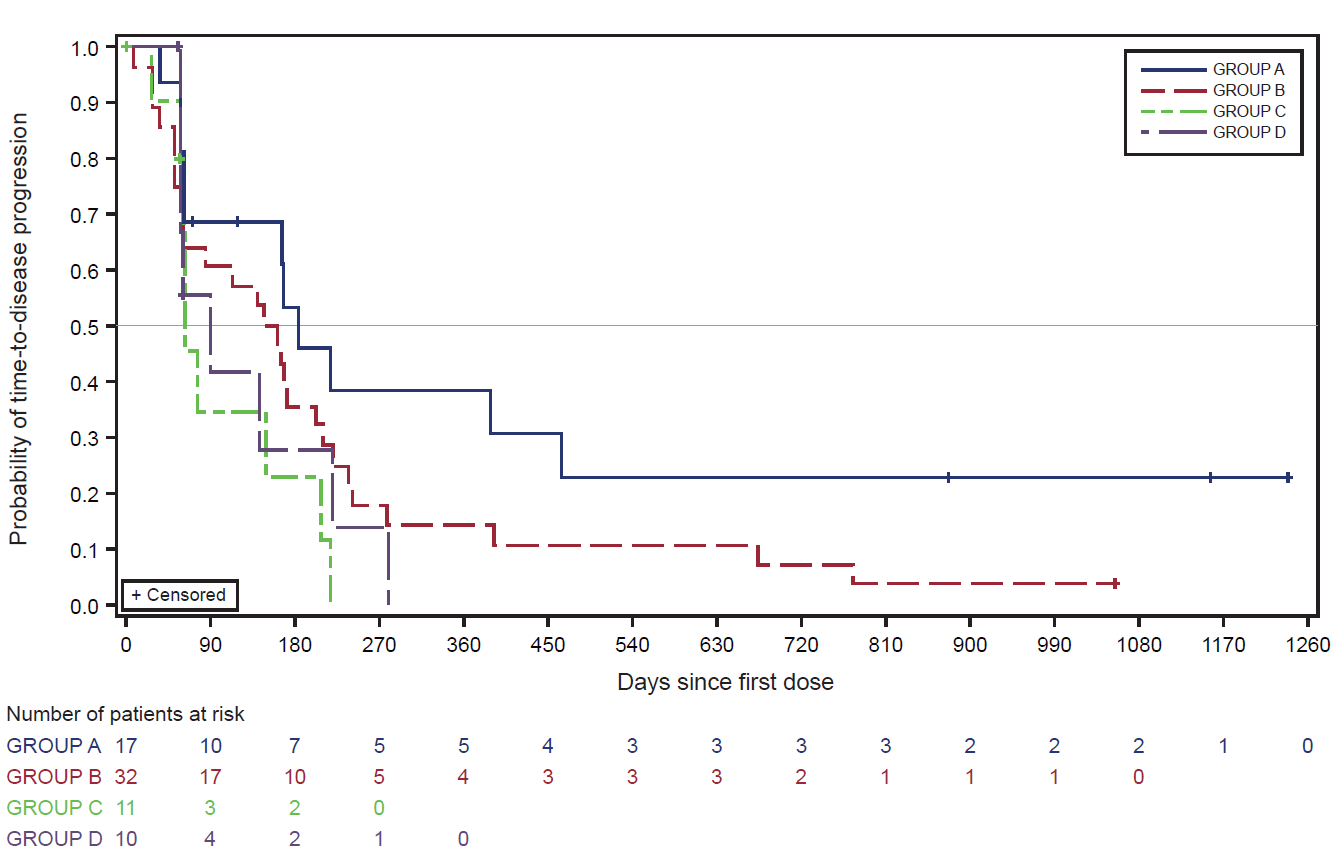

Supplement: Supplementary file 1 — Table S1. Number and percentage of patients per cancer type and overall, detailed per BRCA mutation status, number of prior platinum‐based regimens, and platinum sensitivity.Table S2. Numbers and percentages of patients with measurable disease who received prior platinum‐based regimens, per cancer type and BRCA mutation status, in each study part and overall.Table S3. Relative bioavailability and 90% confidence intervals after single‐dose administration of 200‐mg veliparib‐ER‐C or veliparib‐IR*.Table S4. Geometric mean (% CV) multiple‐dose pharmacokinetic parameters of veliparib‐ER‐C in Part 2 of the study.Table S5. Numbers and percentages of patients who received prior platinum‐based regimens, per study part and in the overall population.Figure S1. Time to disease progression for breast and ovarian carcinoma patients. [file CAM4-7-2360-s001.docx]
